# High expression of CHML predicts poor prognosis of multiple myeloma

**DOI:** 10.7150/jca.34465

**Published:** 2019-10-15

**Authors:** Weilong Zhang, Ling Cao, Xiaoni Liu, Xue He, Ye Zhang, Zuozhen Yang, Ping Yang, Jing Wang, Kai Hu, Xiuru Zhang, Weiyou Liu, Xiaoliang Yuan, Hongmei Jing

**Affiliations:** 1 Department of Hematology, Lymphoma Research Center, Peking University Third Hospital, Beijing, 100191, China; 2 Gannan Medical University, Ganzhou, 341000, China; 3 Department of Respiratory Medicine, The First Affiliated Hospital of Gannan Medical University, Ganzhou, 341000, China; 4 Department of Pathology, Beijing Tiantan Hospital, Capital Medical University, Beijing, 100070, China; 5 Melbourne School of Population and Global Health, The University of Melbourne, Victoria, 3010, Australia

**Keywords:** CHML, multiple myeloma, prognosis, gene expression profile, cell division

## Abstract

Multiple myeloma is a hematological tumor with a malignant proliferation of myeloma cells. Although the survival time after treatment has improved, the recurrence rate of MM is still high. Choroideremia-like (CHML) protein is essential for the prenylation modification of various Rab proteins and it exerts biological effects on vesicle trafficking and signal transduction. However, little is identified about the relationship between CHML gene and MM. We integrated gene expression profiles of 1907 MM patients (1959 MM samples) from the 7 datasets. The relationship between CHML gene expression level and event-free survival (EFS), overall survival (OS), ISS stage, molecular subtype, relapse, therapy was analyzed. The differential gene exression profile of CHML-high MM group and CHML-low MM group and possible pathway related to CHML were conducted. Our data showed that EFS (*P* < 0.0001) and OS (*P* < 0.0001) in MM patients with high expression of CHML were lower than those with low CHML expression. The gene expression level of CHML was increased in subtypes of MM with poor prognosis, especially in proliferation subtype (*P* < 0.001). Cell division pathway (*P* < 0.01) was high enriched of the differential expressed genes of CHML-high group vs CHML-low group. CHML gene can be considered as an independent factor to evaluate the prognosis of MM. High expression of CHML is associated with poor survival, which is related to cell proliferation and cell division of myeloma cells.

## Introduction

Multiple myeloma is a malignant tumor that affects numbers of people worldwide. Despite several therapeutic interventions are available, it still remains a poor clinical prognosis and high relapse rate 1-3. The international staging system (ISS) is considered as a canonical prognostic staging criterion for MM. The ISS divides MM into three phases, ISS I (serum β2-microglobulin levels <= 3.5 mg / L and serum albumin >= 35 g / L), ISS II (excluding the ISS I and ISS III) and ISS III (serum β2-microglobulin levels >= 5.5 mg / L) 4. The genome of MM presents a variety of complexity and genetic instability 5-7. The translocation / cyclin D (TC) typing standard was based on the immunoglobulin H (IgH) translocation and the expression of cyclin D (CCND) genes. The TC criteria divide MM into 8 groups, including 11q13, 6p21, 4p16, maf, D1, D1+D2, D2, and none 8. From the perspective of biogenetics, MM can be divided into hyperdiploid and non-hyperdiploid, and hyperdiploid patients have higher survival level than non-hyperdiploid patients 9-12. According to the current University of Arkansas for Medical Science (UAMS) classification, MM is divided into seven subtypes. Including proliferation (PR), low bone disease (LB), MMSET (MS), hyperdiploid (HY), CD-1, CD-2 and MAF / MAFB (MF) 13. Based on this classification standard, Broyl A et al. added three categories, including cancer testis antigens (CTA), nuclear factor kB (NF-kB) and PRL3 14. Overall, the PR subtype is confirmed as a poor prognosis and short-term survival label of MM 13-15.

The International Myeloma Working Group (IMWG) has added three new biomarkers to the newly updated diagnostic criteria for MM 16, which reflects the importance of MM biomarkers and need further developments. Although numbers of studies have been conducted on MM, the diagnosis of MM still principally depends on the clinicopathological features. Therefore, more powerful biomarkers are needed to be studied in terms of the diagnosis and prognosis of MM, and provide better options for the treatment of MM in the future. In particular, high-risk asymptomatic MM patients are more likely to be highly malignant. Therefore, it is of great importance to detect MM at an early stage and provide treatments to the patients as soon as it has been confirmed.

The CHML gene is located on chromosome 1q43 17. CHML has a high degree of sequence similarity to Choroideremia (CHM) and can replace CHM binding to Rab proteins 18. It has been reported that in the early stage of invasive urothelial carcinoma, CHML gene is significantly overexpressed in the cytoplasm mainly around the nucleus 19. In addition, CHML may also be a susceptibility gene for asthma and was speculated to play a role by affecting the prenylation of specific Rab protein 20. We found CHML as a meaningful gene which is related to MM by analyzing the gene expression profiles of a large number of MM patients. And our investigation verified that the high expression of CHML gene is harmful for the survival of MM patients and likely associated with cell proliferation and division.

## Materials and Methods

### Data source

In our study, gene expression microarrays of 1907 MM patients were derived from Gene Expression Omnibus database, including datasets GSE24080 (559 MM patients) 21, GSE9782 (264 MM patients) 22, GSE19784 (311 samples) 23, GSE83503 (585 MM patients) 24, GSE82307 (33 MM patients) 25, GSE19554 (19 MM patients) 25, and GSE39754 (136 MM patients) 26. The subjects selected in our study were MM patients and were given the corresponding information such as clinical features, treatment response or related biochemical examinations, and had published high throughput gene expression data. This research was in accordance with the Declaration of Helsinki.

### Microarray analysis

All microarray datasets were analyzed and systematically screened for the significant aberrant expression gene and which could be a prognostic assessment. The different expression profile were conducted from CHML-low group vs CHML-high group were also analyzed and ranked by foldchange values (log2,* P* < 0.05 must be satisfied).

### Gene Ontology (GO) analysis

Use the DAVID to analyze the 559 samples in the dataset GSE24080 and find out the enrichment pathways for different expressed genes between CHML-low group and CHML-high group^27^. The results were ranked by the P value (-log10).

### Statistical analysis

Statistical analysis was performed by R software v3.1.3 (ggplot2 and survminer package). The Kaplan-Meier method and log-rank test were used for survival analysis. Descriptive statistics were presented in the form of mean and standard deviation. *P* < 0.05 was defined as statistically significant.

## Results

### The expression of CHML is higher in the poor ISS stage of MM

**Figure 1:**
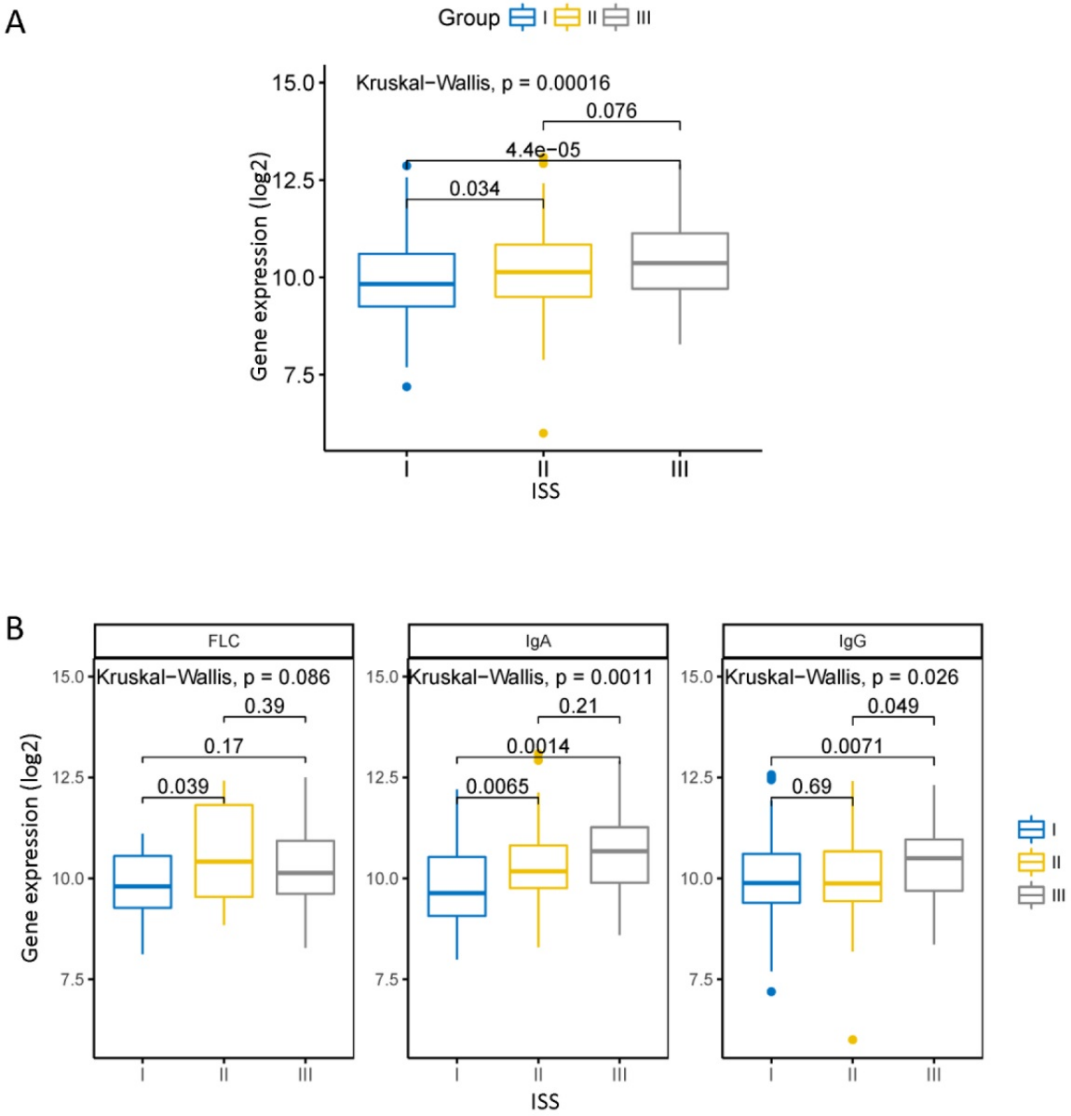
CHML gene expression in the different ISS clinical stages of MM. A, The expression of CHML in different ISS phases of MM. B, The expression of CHML in different ISS clinical stages in various subtypes (FLC group, IgA group, and IgG group) of MM. The Y-axis represents the level of CHML gene (log2), and the X-axis represents the ISS clinical stage of the MM.

The ISS is a widely used staging standard that divides MM into three phases 16. We compared the expression of CHML in different ISS stage in dataset GSE24080. There was a statistically significant increase of the level of CHML from ISS I to ISS III (Fig. 1A, Kruskal-Wallis test, *P* = 0.00016). In each of the monoclonal immunoglobulin group (except free light chain [FLC] group) of MM, the expression of CHML is obviously different among each ISS stage (Fig. 1B, Kruskal-Wallis test, FLC:* P* = 0.066, IgA: *P* = 0.0011, IgG: *P* = 0.026). The levels of CHML in FLC group and immunoglobulin A (IgA) group increased significantly between ISS I and ISS II (*P* < 0.05) but not between ISS I and ISS III (*P* > 0.05). However, it is different in the immunoglobulin G (IgG) group between ISS I and ISS III (*P* < 0.05). The level of CHML in IgG type did not show significant increase between ISS I and ISS II (*P* > 0.05), but it is evident between ISS II and ISS III (*P* < 0.05). Overall, the expression of CHML gradually increased with the ISS stage from low to high.

### Different expression of CHML in different molecular types of MM

**Figure 2:**
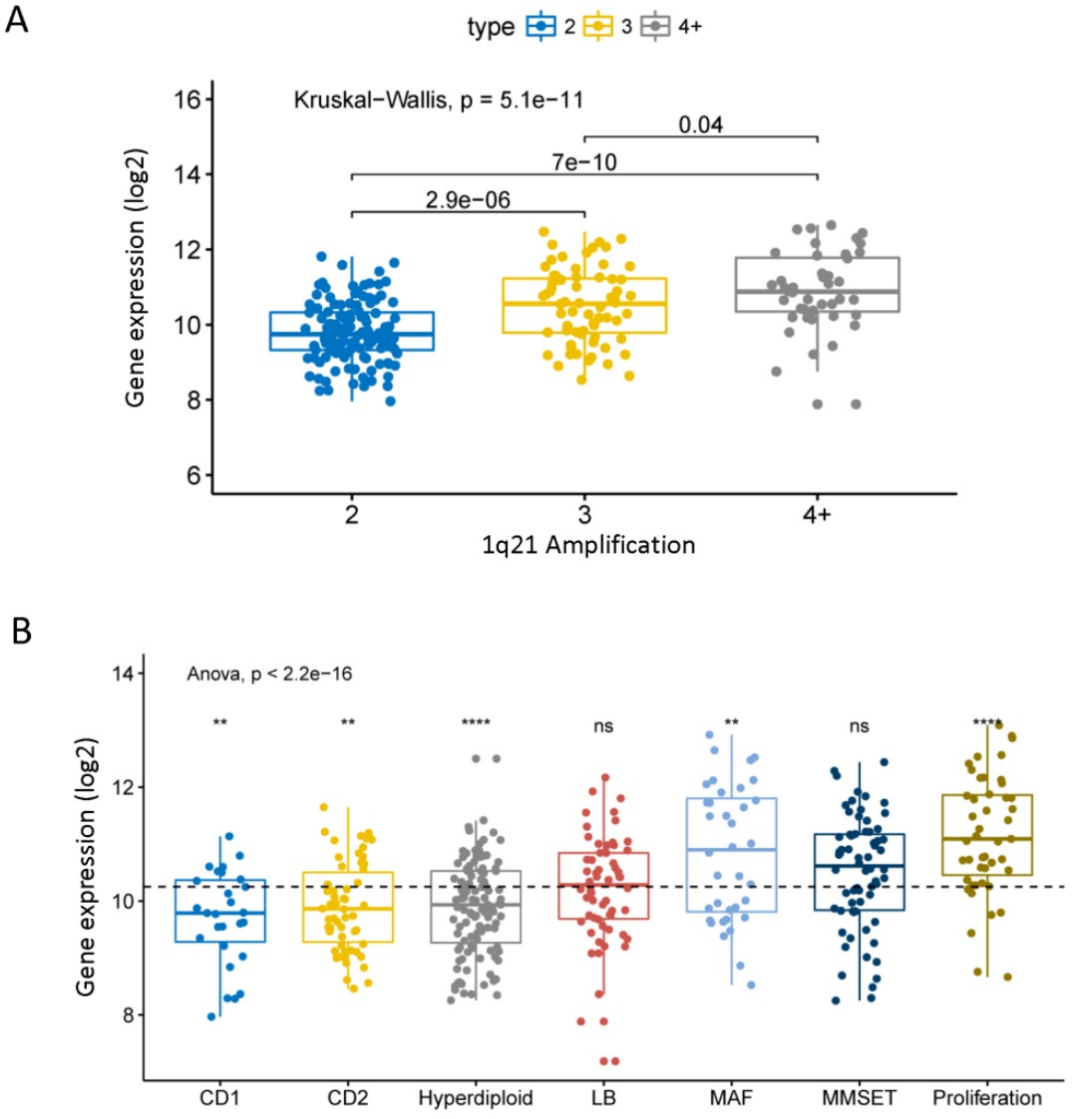
The expression of CHML in different molecular subtypes of MM. A, The expression of CHML at different amplification levels of 1q21 in MM. B, The level of CHML in seven different molecular subtypes of MM. Different icons indicate different statistical saliency: ns: *P* > 0.05, *: *P* <= 0.05, **: *P* <= 0.01, ***: *P* <= 0.001, ****:* P* <= 0.0001. The dotted line represents the average of all values. The Y-axis represents the CHML expression (log2), and the X-axis represents the subtype of MM.

Chromosome 1q21 amplification is a very important cytogenetic abnormal event of MM and is associated with the progression and poor prognosis of MM 28. We found that the expression level of CHML significantly increased with the 1q21 amplification in dataset GSE24080 (Fig. 2A, *P* = 5.1e-11). The data shows that the expression of CHML in different molecular subtypes of MM is roughly divided into two groups. As shown in Fig. 2B (Anova test,* P* = 2.2e-16), the expression levels of CHML gene in seven molecular subtypes are significantly different. The expression levels of CHML are higher in MF, MS, and PR groups (PR group is the most obvious,* P* < 0.001), however, the expression levels of CHML gene in the other four molecular subtypes (CD1, CD2, HY and LB) are lower (HY group is particularly noticeable,* P* < 0.0001). In addition, another dataset GSE19784 of 311 MM patients was analyzed (Fig. S1 and Table S1, *P* = 6.1e-14, Anova test). Similarly, the expression of CHML in the PR group is significantly increased, whereas the CHML levels in CTA and NF-kB groups are decreased. And there is no significant difference in other groups (*P* > 0.05).

### CHML predictes poor survival and relapse of MM

**Figure 3:**
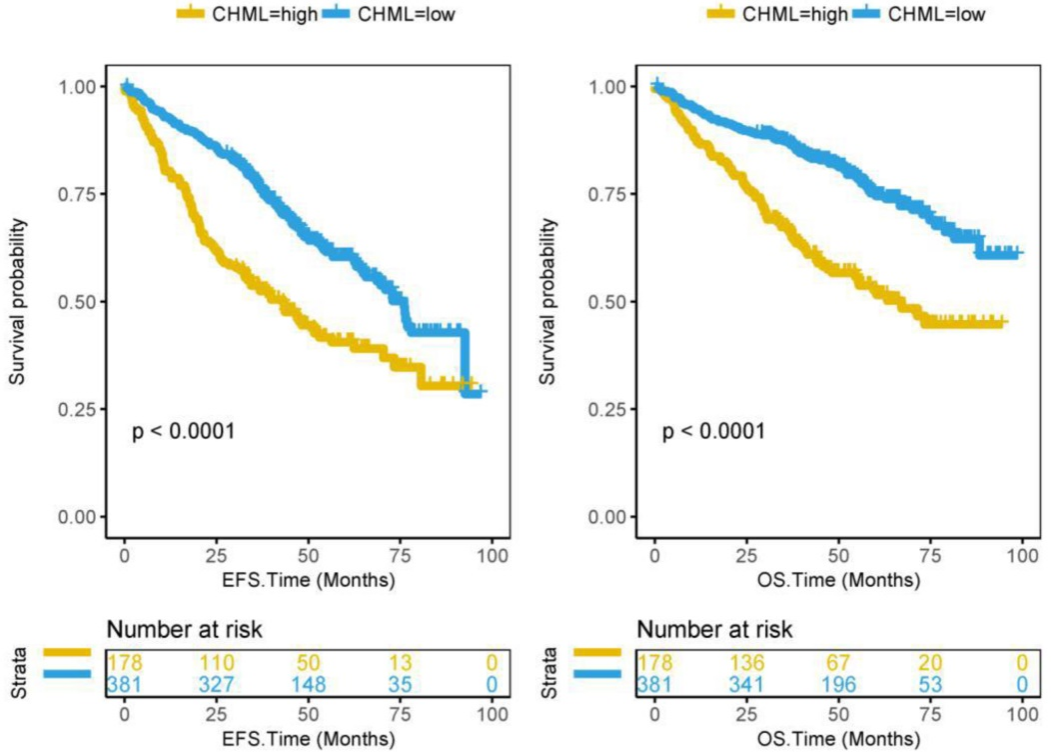
Survival analysis of CHML gene in 559 MM patients from dataset GSE24080. Kaplan-Meier Curves for Event-free survival (left) and Overall survival (right) in 559 MM patients. Log-rank test was used. The Y-axis represents survival probability, and the X-axis represents survival time (months).

From the previous results, it can be seen that CHML is always related to bad events of MM. It is not difficult to speculate that high expression of CHML is a predictor of poor prognosis of MM. Subsequently, we confirmed this speculation by the survival analysis of 559 patients in dataset GSE24080. 559 MM patients were divided into CHML-high group (178 patients) and CHML-low group (381 patients) according to the expression level of CHML. The survival time of MM is generally shorter. Kaplan-Meier curves showed that event-free survival (*P* < 0.0001) and overall survival (*P* < 0.0001) are significantly lower in the CHML-high group than in the CHML-low group (Fig. 3, log-rank test). The same result was also shown in another dataset GSE9782 that included 264 MM patients (Fig. S2, *P* < 0.0001, log-rank test), which further confirmed that MM patients with high expression of CHML had worse survival. In the dataset GSE83503 containing 585 MM patients, the relapse group has a high expression of CHML (Fig. S3 and Table S2, *P* = 0.0002, unpaired t test, two sided). In other words, MM patients with high expression of CHML are more likely to relapse than low expression of CHML.

### The expression of CHML is an independent prognostic factor in MM

559 MM patients in GSE24080 were analyzed by Cox regression analysis (Table 1). The result shows the hazard ratios (HR) and 95% confidence interval (95%CI) of CHML gene (>= 10.55) in EFS (HR: 1.86, 95%CI = 1.43 to 2.42, *P* = 4.34e-6) and OS (HR: 2.32, 95%CI = 1.69 to 3.17, *P* = 1.44e-7), respectively. Albumin (ALB, >= 35 g/l) and haemoglobin (HGB, >= 100 g/l) are favorable factors for MM patients, and their HRs are less than one in both EFS (ALB: 0.87 [95%CI = 0.61 to 1.23, *P* = 0.432]; HGB: 0.78 [95%CI = 0.58 to 1.05, *P* = 0.105] ) and OS (ALB: 0.70 [95%CI = 0.47 to 1.04, *P* = 0.0784], HGB: 0.89 [95%CI = 0.62 to 1.27, *P* = 0.508] ), even if they are not significant. And the HRs of the other three unfavorable factors are higher than one, including beta-2 microglobulin (B2M, >= 3.5 mg/l, EFS: 1.43 [95%CI = 1.05 to 1.95, *P* = 0.0218]; OS: 1.67 [95%CI = 1.15 to 2.44, *P* = 0.00749] ), number of magnetic resonance imaging-defined focal lesions (skull, spine, pelvis) (MRI, >= 3 focal lesions, EFS: 1.47 [95%CI = 1.13 to 1.93, *P* = 0.00485]; OS: 1.99 [95%CI = 1.42 to 2.79, *P* = 7.04e-5] ) and bone marrow biopsy plasma cells (BMPC, >= 35%, EFS: 1.43 [95%CI = 1.04 to 1.96, *P* = 0.029]; OS: 1.35 [95%CI = 0.91 to 2.00, *P* = 0.135] ). It has been demonstrated that CHML can be considered as an independent predictor of clinical prognosis of MM, which predicts poor survival of MM (HR > 1, *P* < 0.05).

### The baseline characteristics of patients between CHML-low group and CHML-high group

We compared the baseline and clinicopathological characteristics between CHML-high group and CHML-low group in dataset GSE24080 (Table 2). Lactate dehydrogenase (LDH,* P* < 0.001), HGB (*P* < 0.001), aspirate plasma cells (ASPC, *P* = 0.01) and BMPC (*P* = 0.004) are significantly different between CHML-low group and CHML-high group. However, there is no statistically significant difference in age (*P* = 0.108), sex (*P* = 0.206), race (*P* = 0.719), isotypes (P = 0.89), B2M (*P* = 0.065), CRP (C-reactive protein, *P* = 0.06), creatinine (CREAT, *P* = 0.49), ALB (*P* = 0.059) and MRI (*P* = 0.317). Three “bad tags” of MM (LDH, ASPC and BMPC) in CHML-high group show significant increase compared to the CHML-low group. Whereas, HGB, a “good tag” of MM, shows significant decrease in CHML-low group. Although there is no statistical significance, the mean value of other several unfavorable factors (age, B2M, CRP, CREAT, and MRI) of MM in CHML-high group are higher compared to those in CHML-low group, however, one favorable factor (ALB) is lower in CHML-high group. There was no significant correlation between the expression level of CHML and the baseline characteristics of MM patients in dataset GSE9782 (Table S3, *P* > 0.05).

### The level of CHML did not change significantly before and after treatment in MM patients

In order to understand whether the level of CHML was changed in MM patients before and after the therapy, 238 patients who were treated with either bortezomib (MM patients) or dexamethasone (MM patients) in dataset GSE9782 were tested by U133A and U133B array respectively (totally 476 arrays). Unfortunately, the results show that the levels of CHML are not significantly different in each stratification of the post-treatment response (Fig. S4A and B left, *P* > 0.05), however, patients in dexamethasone treatment group show a statistical significance (Fig. S4B right, *P* = 0.013). Only low expression of CHML in MR group treated with bortezomib by the second detection method is meaningful (Fig. S4B left, *P* < 0.05). In another dataset (GSE39754), 136 newly diagnosed MM samples were analyzed. The level of CHML is still not significantly changed in the four groups of response after treatment (Fig. S5, *P* = 0.2).

In addition, there is no significant difference in the expression level of CHML before and after relapse in each of the 33 MM patients in dataset GSE82307 (Fig. S6A and Table S4, Wilcoxon test, *P* = 0.25). Similarly, the level of CHML has no significant change before and after treatment (first chemotherapy) in each of the 19 MM patients in GSE19554 (Fig. S6B, *P* = 0.22).

### CHML gene is associated with cell division and proliferation

**Fig 4:**
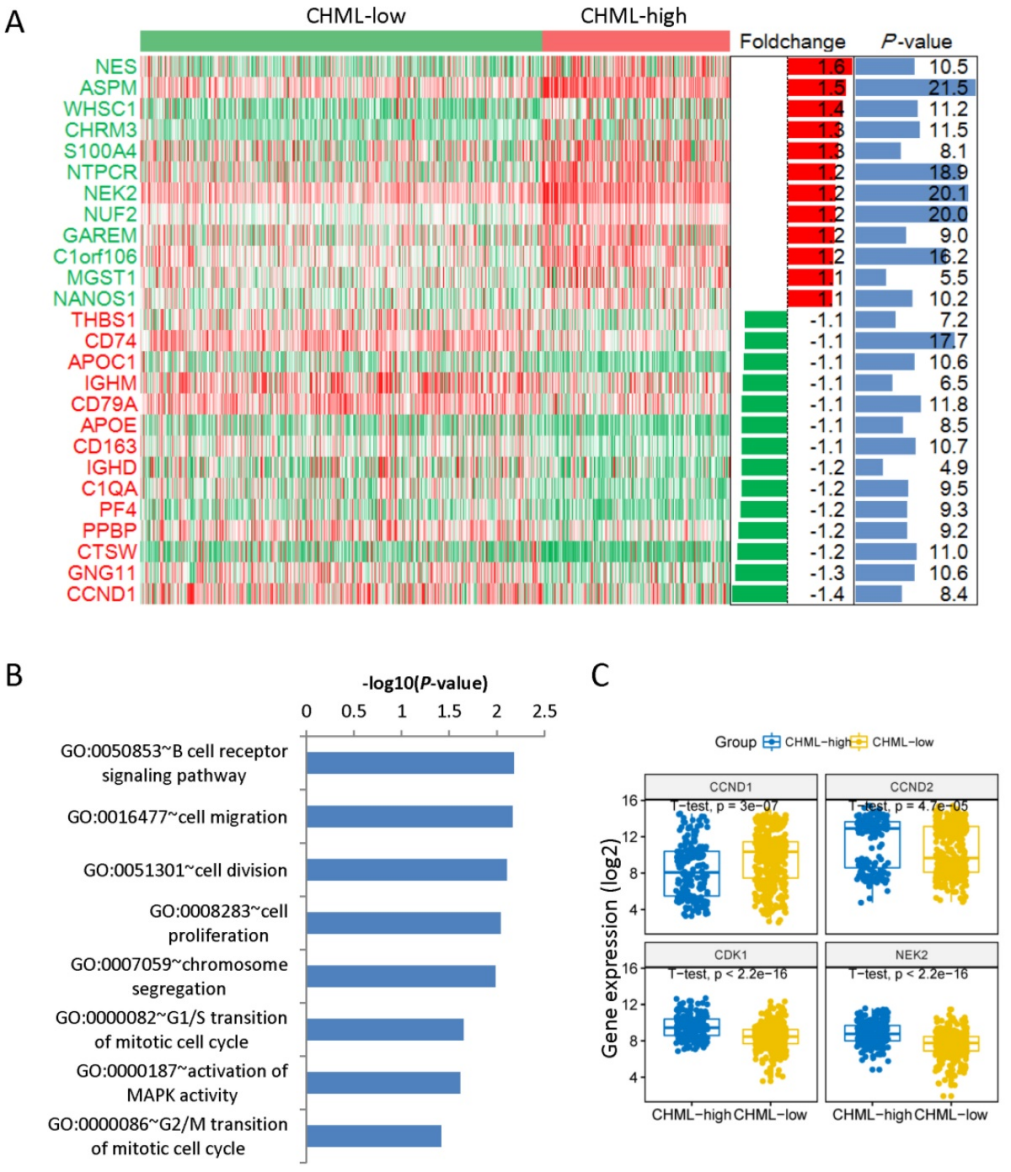
Different expression of genes and enrichment pathways. A, Different expression genes between CHML-low group and CHML-high group. The heat map showed top12 up-regulated and top12 down-regulated genes (sorted by foldchange). The right side is the corresponding foldchange (log2) and *P* value (-log10). Red and green represent high and low expression of the CHML gene, respectively. B, The main enriched pathways for different expression genes (sorted by *P* value). C, The level of the four different expression genes in the cell division pathway was compared between CHML-low group and CHML-high group, respectively.

We have found 40 down-regulated and 33 up-regulated genes comparing the gene expression profiles between these two groups. The heat map shows only top12 up-regulated and top12 down-regulated genes (Fig. 4A). NES (foldchange [log2] = 1.6, *P* < 0.05) is the top 1 up-regulated gene and CCND1 (foldchange [log2] = -1.4, *P* < 0.05) is the top 1 down-regulated gene. We analyzed the main enriched pathways of these differentially expressed genes. The first one is B cell receptor signaling pathway, followed by cell migration, cell division and cell proliferation (Fig. 4B). Then analyzed the four different expression genes in the pathway of cell division between CHML-high group and CHML-low group (Fig. 4C, unpaired t test, two sided). The expression of genes of CCND2 (*P* = 4.7e-5), CDK1 (*P* < 2.2e-16) and NEK2 (*P* < 2.2e-16) are obviously increased in CHML-high group, whereas CCND1 gene (*P* = 3e-7) is evidently reduced. Combined with the previously described expression of CHML among seven different molecular subtypes of MM, CHML may play an important role in tumor cell proliferation and division.

## Discussion

MM is an incurable tumor with plasma cells malignant proliferation and abnormal secretion of immunoglobulins 29-31. The great complexity and instability of genome exert heavy burdens to improve the efficacy of current therapies and reduce the relapse rate of MM. The knowledge of the biological effects of related molecules in MM contributes to determining appropriate therapies and improving the outcome for MM patients, especially for asymptomatic high-risk MM patients. Therefore, further researches need to be conducted on the pathogenesis and therapies of MM from the perspective of genome and molecules. CHML, also known as Rab escort protein 2 (REP2), is one of the key factors for the prenylation of various Rab proteins. Numerous studies have demonstrated the positive effects of prenylation of Rab protein on cell proliferation, survival, and division 32-34. In our study, we have shown that the expression of CHML gene is related to MM.

In this large sample-based study (1907 MM patients), the results indicates that high expression of CHML predicts worse survival. This is consistent with the expression pattern of CHML gene in invasive urothelial carcinomas that highly expressed CHML is similarly indicates a low survival level 19. The worse the clinical stage of MM, the higher the level of CHML. In other words, the level of CHML is parallel to the severity of MM. In the survival analysis, it is obvious that the survival level of MM patients in CHML-high group is evidently lower compared with patients in CHML-low group. CHML gene can be used as an independent factor to determine the prognosis. And recurrent MM patients show high expression level of CHML. However, the level of CHML in the post-relapse MM do not increase further as it is not significant different. There is still have no change in the expression of CHML before and after the treatment, which indicates the genetic stability is not easily affected by the treatment of MM.

From the CHML-low group to the CHML-high group, the level of unfavorable clinical pathology (such as LDH, CRP, BMPC etc.) of MM is increased, while the level of favorable one (HGB) is lessened. In our study, it is noteworthy that the number of patients remains the highest in IgG type and the lowest in IgD type, no matter the patients in CHML-high group or CHML-low group. Compared with other types, the IgD type often represented as a poor clinical prognosis of MM 35, 36. A consistent trend of CHML expression is identified in seven different molecular subtypes (UAMS classification) of MM. The expression level of CHML is increased in subtypes MF, MS and PR, and is reduced in subtypes CD1, CD2, HY and LB. Coincidentally, MF, MS, and PR were high-risk groups for MM, while other subtypes (CD1, CD2, HY and LB) were associated with good survival 13, 15, 37.

Another focus is on the potential biological function of the CHML gene in MM. CHML is highly expressed in PR molecular subtype (both in dataset GSE24080 and GSE19784) of MM and is positively correlated with three genes (CCND2, CDK1, and NEK2) that regulate cell division. This indicates that the CHML gene may be involved in the regulation of proliferation and division of myeloma cells in MM. As mentioned above, existing related studies have shown that the main role of CHML gene is to regulate the function of Rab protein, and it has also been reported that Rab protein can affect myeloma cells. So it is speculated that CHML may regulate the proliferation and division of myeloma cells by acting on Rab protein, but this needs to be further confirmed.

Moreover, it should be noted that CCND1 activation is a favorable prognostic indicator of MM 38, but it is still debated 39, 40. And as a controversial gene of MM, CCND1 is reduced in high expression of CHML. According to the preceding description, the increased expression of CHML is closely associated with unfavorable factors for MM, it is speculated that CCND1 may be a favorable prognostic factor for MM in our study samples.

There are some limitations in study. Although we report the role of CHML gene in MM, the molecular mechanism is still unclear. Whether CHML gene is closely related to known driver genes or key pathways of MM, and whether CHML gene combined with known biomarkers can be better for the diagnosis and staging of MM. If an increase of CHML is detected in the early stage of MM patients, can immediate treatment extend the survival time? In addition, whether chemotherapy regimens can be simplified (avoid over-chemotherapy) for MM patients with low level of CHML. These problems are not solved in this paper, and further relevant experiments are needed to verify.

In conclusion, CHML gene is a new meaningful prognostic factor for MM. High level of CHML predicts poor survival and high recurrence rate in MM patients. And the over expression of CHML gene has a high risk for MM. CHML gene as a “detrimental gene” of MM may play a role in regulating the proliferation and division of myeloma cells. Therefore, further exploration of the biological behavior of CHML in MM may provide certain benefits for the prognosis and treatment of MM in the future.

## Supplementary Material

Supplementary figures and tables.

## Figures and Tables

**Table 1:** Cox regression analysis of CHML expression in 559 MM patients patients in dataset GSE24080.

		95% CI for HR		
	HR	Lower	Upper	*P*-value
EFS				
B2M (>= 3.5 mg/l)	1.43	1.05	1.95	2.18E-02
ALB (>= 35 g/l)	0.87	0.61	1.23	4.32E-01
HGB (>= 100 g/l)	0.78	0.58	1.05	1.05E-01
MRI (>= 3 focal lesions)	1.47	1.13	1.93	4.85E-03
BMPC (>= 35%)	1.43	1.04	1.96	2.90E-02
CHML (>=10.55)	1.86	1.43	2.42	4.34E-06
				
OS				
B2M (>= 3.5 mg/l)	1.67	1.15	2.44	7.49E-03
ALB (>= 35 g/l)	0.70	0.47	1.04	7.84E-02
HGB (>= 100 g/l)	0.89	0.62	1.27	5.08E-01
MRI (>= 3 focal lesions)	1.99	1.42	2.79	7.04E-05
BMPC (>= 35%)	1.35	0.91	2.00	1.35E-01
CHML (>=10.55)	2.32	1.69	3.17	1.44E-07

CI, confidence interval; HR, hazard ratio. EFS, Event-free survival time (months), the date of definition is from registration to death of any reason, disease progression or recurrence, or checked at the last contact; OS, Overall survival time (months), the date of definition is from registration to death of any reason or checked at the last contact. B2M, Beta-2 microglobulin (mg/l); ALB, Albumin (g/l); HGB, Haemoglobin (g/l); MRI, Number of magnetic resonance imaging (MRI)-defined focal lesions (skull, spine, pelvis); BMPC, Bone marrow biopsy plasma cells (%); CHML, Choroideremia-like.

**Table 2:** The baseline characteristics of MM patients in dataset GSE24080 between CHML-low group and CHML-high group.

		CHML-low	CHML-high	P-value
n		381	178	
AGE (mean (sd))		56.74 (9.58)	58.12 (9.16)	0.108
SEX (%)	female	144 (37.8)	78 (43.8)	0.206
	male	237 (62.2)	100 (56.2)	
RACE (%)	other	44 (11.5)	18 (10.1)	0.719
	white	337 (88.5)	160 (89.9)	
ISOTYPE (%)	FLC	59 (15.5)	25 (14.0)	0.89
	IgA	85 (22.3)	48 (27.0)	
	IgD	2 ( 0.5)	1 ( 0.6)	
	IgG	218 (57.2)	95 (53.4)	
	Nonsecretory	4 ( 1.0)	2 ( 1.1)	
	Unknown	13 ( 3.4)	7 ( 3.9)	
B2M (mean (sd))		4.44 (5.24)	5.34 (5.59)	0.065
CRP (mean (sd))		10.38 (20.59)	14.31 (27.30)	0.06
CREAT (mean (sd))		1.30 (1.24)	1.38 (1.35)	0.49
LDH (mean (sd))		161.77 (51.71)	193.81 (85.20)	**<0.001**
ALB (mean (sd))		4.08 (0.57)	3.98 (0.60)	0.059
HGB (mean (sd))		11.44 (1.77)	10.85 (1.83)	**<0.001**
ASPC (mean (sd))		40.84 (23.97)	46.65 (24.69)	**0.01**
BMPC (mean (sd))		44.17 (26.34)	51.06 (25.59)	**0.004**
MRI (mean (sd))		10.60 (14.52)	11.97 (14.55)	0.317

n, number of patients; sd, Standard deviation. CRP, C-reactive protein (mg/l); CREAT, Creatinine (mg/dl); LDH, Lactate dehydrogenase (U/l); ALB, Albumin (g/dl); HGB, Haemoglobin (g/dl) ; ASPC, Aspirate plasma cells (%). The statistical method used for SEX, RACE and ISOTYPE is Fisher's exact probability test, and others is unpaired t test (two sided).
