# Supplementary material for: High expression of CHML predicts poor prognosis of multiple myeloma
Source: J Cancer. 2019 Oct 15;10(24):6048–56. doi: 10.7150/jca.34465 (PMC6856588; doi:10.7150/jca.34465)

Fig. S1. The expression of CHML in different molecular subtypes of 311 MM patients in dataset GSE19784. The dotted line represents the average of all values. The Y-axis represents the CHML expression (log2), and the X-axis represents the subtype of MM.

Fig. S2. Survival analysis of CHML gene in dataset GSE9782 that included 264 MM patients. Kaplan-Meier Curves for overall survival in 264 MM patients. Log-rank test was used. The Y-axis represents survival probability and the X-axis represents survival time (days).

Fig. S3. The relationship between CHML gene expression and relapse of 585 MM patients in dataset GSE83503. The Y-axis represents the CHML expression (log2), and the X-axis represents relapse or not.

Fig. S4. The expression levels of CHML in different responses after different treatments of 238 MM patients in dataset GSE9782. (A) The expression level of CHML (U133A array) in various treatment responses after treated with bortezomib (left) and dexamethasone (right) in 238 MM patients, respectively. (B) The expression level of CHML (U133B array) in various treatment responses after treated with bortezomib (left) and dexamethasone (right) in 238 MM patients, respectively. Treatment responses: complete response (CR), partial response (PR), minimal response (MR), no change (NC), or progressive disease (PD). Different icons indicate different statistical saliency: ns: $P > 0.05$, *: $P <= 0.05$, **: $P <= 0.01$, ***: $P <= 0.001$, ****: $P <= 0.0001$. The dotted line represents the average of all values. The Y-axis represents the CHML expression (log2), and the X-axis represents post-treatment response.

Fig. S5. The expression of CHML in different responses after treatment of 136 MM patients in dataset GSE39754. All patients received Vincristine, Adriamycin, and Dexamethasone (VAD) induction therapy followed by Autologous Stem Cell Transplant (ASCT). Treatment responses: Complete Response (CR); Very Good Partial Response (VGPR); Partial Response (PR); No Response, Stable disease (NR); No Response, Progressive disease (Prog). Different icons indicate different statistical saliency: ns: $P > 0.05$, *: $P <= 0.05$, **: $P <= 0.01$, ***: $P <= 0.001$,

****: *P* <= 0.0001. The dotted line represents the average of all values. The Y-axis represents the CHML expression (log2), and the X-axisa represents post-treatment response.

Fig. S6. Comparison of CHML levels in each MM patient before and after relapse or treatment. (A) The levels of CHML before and after recurrence of 33 MM patients in dataset GSE82307. (B) The levels of CHML before and after treatment of 19 patients in GSE19554. The baseline is the pre-treatment specimen, and the pre-1st is the specimen after the first chemotherapy.

Table S1 The baseline characteristics of MM patients based on clinical stage and molecular subtype in datatset GSE19784.

Characteristics	Level	CD1	CD2	CTA	HY	MF	MS	Myeloid	NFKB	PR	SOCS3/PRL3	P-value
n		13	34	22	77	32	33	39	37	15	9	
ISS (%)	I	4 (30.8)	17 (50.0)	9 (40.9)	29 (37.7)	10 (31.2)	10 (30.3)	17 (43.6)	7 (18.9)	5 (33.3)	6 (66.7)	NA
	II	4 (30.8)	7 (20.6)	8 (36.4)	26 (33.8)	9 (28.1)	7 (21.2)	10 (25.6)	12 (32.4)	4 (26.7)	1 (11.1)	
	III	5 (38.5)	6 (17.6)	5 (22.7)	18 (23.4)	10 (31.2)	13 (39.4)	5 (12.8)	15 (40.5)	4 (26.7)	1 (11.1)	
	Unknown	0 (0.0)	4 (11.8)	0 (0.0)	4 (5.2)	3 (9.4)	3 (9.1)	7 (17.9)	3 (8.1)	2 (13.3)	1 (11.1)	
CHML (mean (sd))		7.51 (2.12)	8.64 (1.14)	5.79 (2.46)	7.58 (1.54)	8.44 (1.73)	8.70 (2.10)	7.81 (1.29)	6.33 (2.07)	9.49 (1.69)	8.01 (1.54)	<0.001

The statistical method used for CHML is Anova test.

Table S2. The baseline characteristics of MM patients based on relapse in dataset GSE83503.

Characteristics	Level	relapse-	relapse+	P-value
n		182	403	
relapse (%)	relapse-	182 (100.0)	0 (0.0)	<0.001
	relapse+	0 (0.0)	403 (100.0)	
death (%)	No	182 (100.0)	247 (61.3)	<0.001
	Yes	0 (0.0)	156 (38.7)	
CHML (mean (sd))		4.36 (0.49)	4.54 (0.57)	<0.001

Unpaired t test (two sided) was used for CHML. Fisher's exact probability test was used for relapse and death.

Table S3. The baseline characteristics of MM patients in datatset GSE9782 between CHML-low group and CHML-high group.

Characteristics	Level	CHML-low	CHML-high	P-value
n		74	190	
Sex (%)	Female	27 (36.5)	78 (41.1)	0.576
	Male	47 (63.5)	112 (58.9)	
Race (%)	Asian/Pacific Islander	1 (1.4)	8 (4.2)	0.467

	Black	8 (10.8)	14 (7.4)	
	Hispanic	1 (1.4)	1 (0.5)	
	Other	0 (0.0)	2 (1.1)	
	White	64 (86.5)	165 (86.8)	
Age (mean (sd))		60.24 (11.49)	60.18 (9.67)	0.966
ISOTYPE (%)	FLC	10 (13.5)	41 (21.6)	0.076
	IgA	20 (27.0)	34 (17.9)	
	IgG	39 (52.7)	111 (58.4)	
	IgG/IgA	1 (1.4)	0 (0.0)	
	Nonsecretory	2 (2.7)	2 (1.1)	
	Unknown	2 (2.7)	2 (1.1)	
B2M (mean (sd))		5.45 (7.84)	7.47 (13.74)	0.287
CRP (mean (sd))		11.66 (20.84)	16.48 (31.36)	0.292
ALB (mean (sd))		36.50 (7.94)	36.93 (6.34)	0.659
TC (%)	11q13	4 (5.4)	28 (14.7)	NA
	4p16	9 (12.2)	34 (17.9)	
	D1	35 (47.3)	65 (34.2)	
	D1+D2	5 (6.8)	9 (4.7)	
	D2	14 (18.9)	35 (18.4)	
	D3	3 (4.1)	5 (2.6)	
	MAF	3 (4.1)	10 (5.3)	
	Unknown	1 (1.4)	4 (2.1)	
Treatment (%)	Bortezomib	54 (73.0)	134 (70.5)	0.763
	Dexamethasone	20 (27.0)	56 (29.5)	
Response (%)	CR	5 (6.8)	9 (4.7)	0.393
	IE	9 (12.2)	16 (8.4)	
	MR	7 (9.5)	16 (8.4)	
	NC	18 (24.3)	42 (22.1)	
	PD	12 (16.2)	54 (28.4)	

	PR	23 (31.1)	53 (27.9)	

CR, complete response; MR, minimal response; NC, no change; PD, progressive disease; PR, partial response. The Fisher's exact probability test was used for sex, race, ISOTYPE, TC, treatment and response, and unpaired t test (two sided) was used for others.

Table S4. The baseline characteristics of MM patients in dataset GSE82307.

Characteristics	Level	Presentation	Relapse	P-value
n		33	33	
stage (%)	Presentation	33 (100.0)	0 (0.0)	<0.001
	Relapse	0 (0.0)	33 (100.0)	
gender (%)	female	13 (39.4)	13 (39.4)	1
	male	20 (60.6)	20 (60.6)	
celltype (%)	CD138+	33 (100.0)	33 (100.0)	NA
CHML (mean (sd))		10.73 (1.16)	10.88 (1.24)	0.25

Wilcoxon test was used for CHML. Fisher's exact probability test was used for others.

Supplementary Figure 1

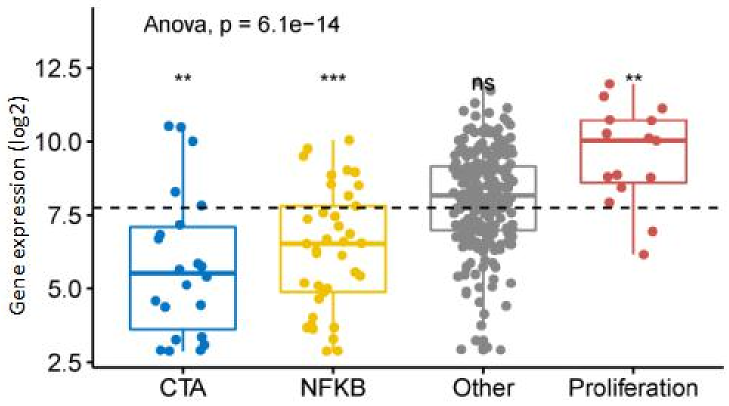

Supplementary Figure 2

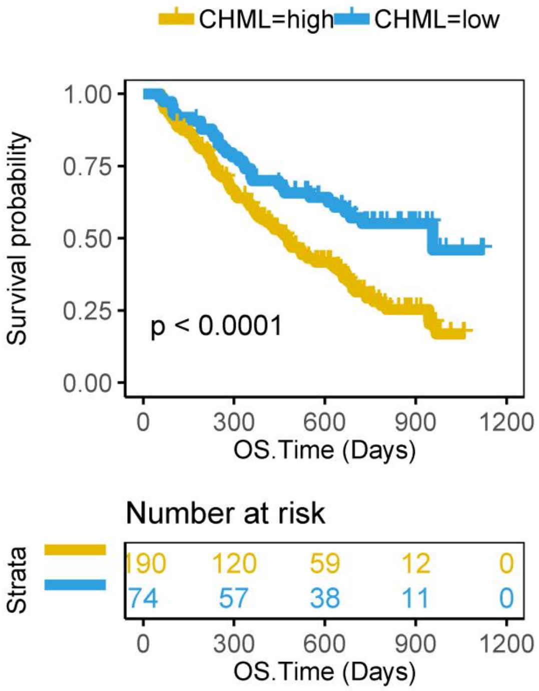

Supplementary Figure 3

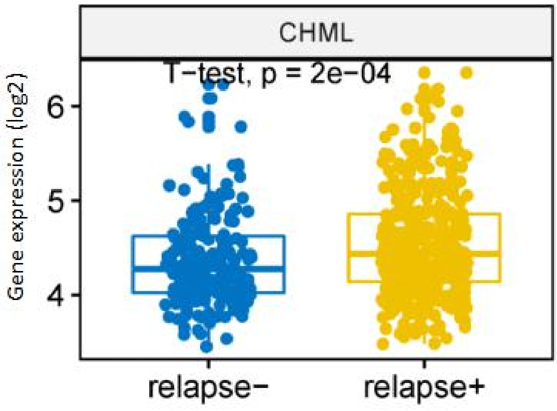

Supplementary Figure 4

A

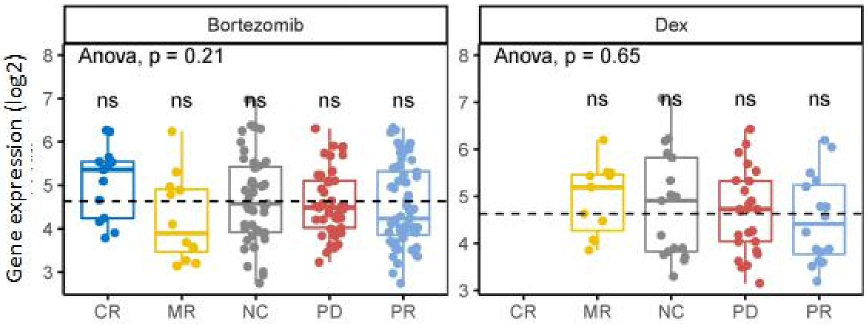

B

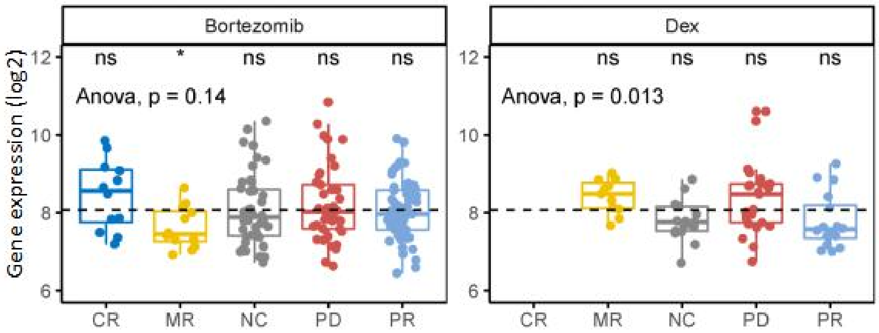

Supplementary Figure 5

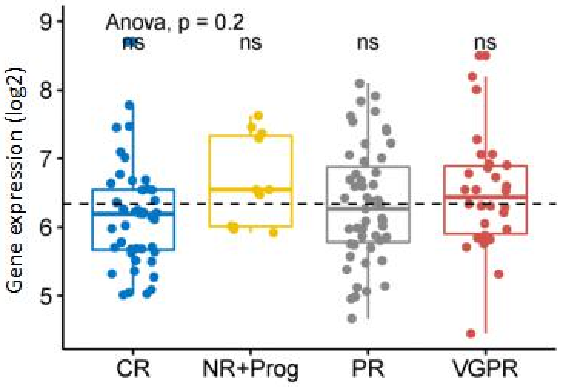

Supplementary Figure 6

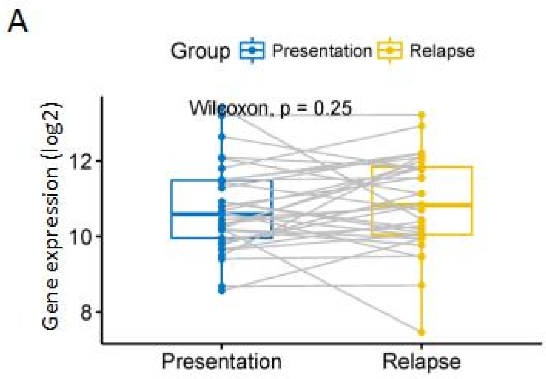

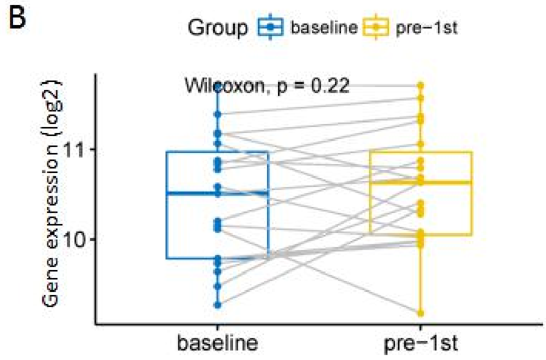

Supplement: Supplementary file 1 — Supplementary figures and tables. [file jcav10p6048s1.pdf]
